# Effects of Nutritional Deprivation and Re-Alimentation on the Feed Efficiency, Blood Biochemistry, and Rumen Microflora in Yaks (*Bos grunniens*)

**DOI:** 10.3390/ani9100807

**Published:** 2019-10-15

**Authors:** Huawei Zou, Rui Hu, Zhisheng Wang, Ali Mujtaba Shah, Shaoyu Zeng, Quanhui Peng, Bai Xue, Lizhi Wang, Xiangfei Zhang, Xueying Wang, Junhua Shi, Fengpeng Li, Lei Zeng

**Affiliations:** 1 Low Carbon Breeding Cattle and Safety Production, University Key Laboratory of Sichuan Province, Animal Nutrition Institute, Sichuan Agricultural University, Chengdu 611130, China; zhwbabarla@126.com (H.Z.); ruitianhu@yeah.net (R.H.); alimujtabashah@sbbuvas.edu.pk (A.M.S.); ifraanwar151@gmail.com (S.Z.); pengquanhui@126.com (Q.P.); xuebai68@163.com (B.X.); wanglizhi08@aliyun.com (L.W.); zxfsicau@foxmail.com (X.Z.); wangxuey_91@163.com (X.W.); mujtaba43@gmail.com (J.S.); fengpli@126.com (F.L.); wangzs1968@gmail.com (L.Z.); 2 Department of Livestock Production, Shaheed Benazir Bhutto University of Veterinary and Animal Sciences, Sakrand 67210, Sindh, Pakistan

**Keywords:** yak, starvation, re-alimentation, compensatory growth, rumen fermentation, rumen microflora

## Abstract

**Simple Summary:**

Yak, the predominant and semi-domesticated livestock on the Qinghai–Tibet Plateau, suffers severe starvation and body weight reduction in the cold season and recovers relatively rapid growth in the warm season every year, because of the harsh highland environment. Rumen microorganisms have critical nutritional and physiological functions for the growth of ruminant, but the strategy of rumen microorganism of yaks to cope with the starvation and re-alimentation challenges and the contributions of rumen microflora to compensatory growth remain unclear. Herein, we investigated the effects of starvation and refeeding on the growth, feed efficiency, blood biochemistry and rumen microbial community as well as functions of yaks. Our results indicated that the rumen microorganism, in part, contributed to yak adaption to starvation and compensatory growth during re-alimentation. Our study is helpful in the understanding and utilization of this natural character of yaks to explore and improve their growth potential.

**Abstract:**

Yak suffers severe starvation and body weight reduction in the cold season and recovers relatively rapid growth in the warm season every year. Herein, we investigated the effects of starvation and refeeding on the growth, feed efficiency, blood biochemistry and rumen microbial community as well as functions of yaks. The results showed that starvation significantly reduced the body weight of yaks. Serum glucose and triglyceride concentrations significantly decreased, and β-hydroxybutyric acid and non-esterified fatty acid levels were significantly increased during the starvation period. Starvation also dramatically inhibited rumen microbial fermentations. Whereas, refeeding with the same diet significantly increased the feed efficiency, nutrient digestibility together with rumen acetate, propionate and microbial protein productions compared with those before starvation. The 16S rDNA sequencing results showed that starvation mainly decreased the ruminal protein-degrading bacteria *Prevotella* and propionate-producing bacteria *Succiniclasticum* populations and dramatically increased the denitrifying bacteria *Thauera* populations. Refeeding reduced the *Euryarchaeota* population and increased propionate-producing bacteria *Succinivibrionaceae UCG-002* and starch-degrading bacteria *Ruminobacter* populations when compared with those before starvation. The predicted microbial metabolic pathways, related to amino acid and starch metabolisms, were also significantly altered during the starvation and refeeding. The results indicated that the rumen microorganisms and their metabolism pathways changed with feed supply, and these alterations in part contributed to yak adaption to starvation and re-alimentation. This study is helpful for enhancing the understanding and utilization of this natural character of yaks to explore and improve their growth potential.

## 1. Introduction

Yaks (*Bos grunniens*) are mainly distributed in the Qinghai–Tibetan Plateau over 3000 m above sea level and are important livestock to the economy of the plateau region by providing milk, meat, and transport for nomadic pastoralists. However, yaks are still mainly raised by natural grazing methods at present. Because of the long cold season (−10–25 °C), plateau grassland is withered, and there is an extreme lack of forage to provide feed for the yaks from October to May of the next year; and the most critical situation is heavy snow disaster, in which the land is covered by thick snow and yaks cannot access any forage. This induces dramatic body weight reduction and mortality. Previous research showed that once food is available again in the short-term warm season, yaks are in a compensatory growth status with higher growth rate and feed efficiency [1]. After long-time evolution, yaks obtained the unique physiological traits to adapt to the extreme high-altitude environment, especially annual starvation and re-alimentation [2]. Our previous work studied the physiological adaption to starvation of yaks, including blood biochemistry, hormone secretions, and metabolic enzyme activity [3], but the alteration of rumen microflora under starvation and refeeding remains unclear.

Starvation, or fasting, is a considerable challenge to many animals and to their gut microorganisms. Previous studies reported that intestinal microorganisms played an essential role in the adaptation and survival of the host during a starved state. For instance, intestinal germ-free mice had higher mortality rates compared to conventional mice during starvation [4]. Germ-free chickens also showed less tolerance to starvation compared to conventional chickens [5]. Presumably, the gut microbiota may provide energy metabolites or alter nitrogen recycling of the host [6]. Additionally, after re-alimentation, animals always have a high efficiency to use dietary nutrition and acquire compensatory growth, which is an important strategy for animals in coping with the fluctuation of feed availability in the natural environment [7]. This natural phenomenon is also widely used in the beef cattle production systems, particularly in reducing overwintering feed-related costs [8] and improving meat characteristics [9]. Currently, increasing studies are focusing on the tissue gene transcriptions [10], nutrient metabolisms [7], as well as endocrine [11] and immunological status [12] of cattle under compensatory growth, but the study on the gastrointestinal microflora contributing to the compensatory growth of ruminants is scarce. McCabe et al. (2015) found rumen propionate production increased in Holstein bulls during refeeding after moderate feed restriction [13], but the alterations of the rumen microbial community under compensatory growth remains unclear. 

The rumen is a unique digestive and absorptive organ of ruminants, which contains abundant and diversified microorganisms. Rumen microbial fermentation is the primary approach to extract dietary nutrition for ruminants. Therefore, this study aimed to investigate the effects of starvation and prolonged refeeding on the feed efficiency, blood biochemistry, rumen fermentation, and rumen microflora of yaks. A more detailed understanding of the contributions of rumen microorganisms to the starvation adaption and compensatory growth of yaks would allow for greater utilization of this natural phenomenon to improve yak production efficiency.

## 2. Materials and Methods

All experimental procedures used in this research were in accordance with the ARRIVE guidelines and the Regulation on the Administration of Laboratory Animals (2017, China State Council). These procedures were approved by the Institutional Animal Care and Use Committee of Sichuan Agricultural University (#SCAUAC201408-3).

### 2.1. Animals, Experimental Design, and Samples

#### 2.1.1. Experiment 1—Effects of Starvation and Refeeding on Yak Growth Performance

This experimental design was divided into two parts. First, the starvation and refeeding models of yaks were evaluated by measuring the growth performance, feed digestibility, blood biochemical indicators, and rumen fermentation of yaks. In experiment part one, a total of twelve 3-year-old healthy male Jiulong yaks (237.79 ± 11.75 kg, mean ± SD) were selected and randomly divided into two groups with six yaks per group, treated with control and starvation, respectively. After a 14-day transitional period, yaks of the starvation group were starved without feed and diet for 7 days (day 0–7). To ensure that yaks were in the post-absorptive condition and did not reach a critical stage of life after being starved, the 7-day starvation time was determined in accordance with our previous work [3]. Then, yaks of the starvation group were refed with the experimental diet (Table 1) ad libitum for another following 7 days (day 8–14). Yaks of the control groups were fed with the same experimental diet ad libitum throughout the trial period. All yaks were penned indoors and provided with diet individually, and allowed free access to water. The body weight of yaks was measured before the morning feed on day 0, 7, and 14 to calculate the average daily gain (ADG). Dietary dry matter intake (DMI) of each yak was recorded every day. The ratio of feed to gain was calculated by the ADG and DMI. Feces samples of each yak were collected using the total fecal collection methods [14] and diet samples were also collected in a digestion trial over day 10 to 13, then stored at −20 °C for determination of the nutrient digestibility. Jugular vein blood was collected before the morning feed on day 0, 7 and 14; then serum was separated after centrifugation (3500 rpm for 20 min) and stored at −20 °C. Rumen fluid was collected using an oral stomach tube [15] at 2 h after morning feeding on day 0, 7, and 14, filtered by four layers of nylon, and stored at −20 °C for analysis of the rumen fermentation parameters.

#### 2.1.2. Experiment 2—Effects of Starvation and Refeeding on Microbial Composition in the Rumen of Yaks

Based on the methods of starvation modelling in experiment one, experiment two was specially designed for deep investigation of the successive alterations of rumen microflora of yaks throughout the feeding, starvation, and prolonged refeeding periods. Another six 3-year-old healthy male Jiulong yaks with similar body weights as the yaks of experiment one were selected. After the 14-day transitional period, all yaks were successively treated with three phases, including normal feeding period (NFP, day 0–7), starvation period (SP, day 8–14) and prolonged refeeding period (RFP, day 15–42), according to a self-controlled trial design [16]. The yaks were individually penned in a controlled indoor environment. During the normal feeding period and refeeding period, yaks were fed with the same diet in experiment one ad libitum. During the starvation period, yaks were starved without receiving any feeding for 7 days [3]. Yaks were allowed free access to water during the experimental period. The rumen fluids were collected by the same method described above on days 7 (NFP), 14 (SP), 21 (RFP1), 28 (RFP2), 35 (RFP3), and 42 (RFP4). Then, rumen fluids were filtered through four layers of nylon and stored at −80 °C for fermentation parameters and 16 s rDNA sequencing analysis.

### 2.2. Experimental Diet

The experimental diet was formulated according to the recommendation of the Chinese Beef Cattle Feeding Standard (NY/T 815-2004) for cattle with body weight of 200 kg and daily gain of 0.8 kg/d. The roughage was distiller’s grains and straw. The ratio of concentrate to roughage was 35:65 (dry matter basis) (Table 1).

### 2.3. Nutrients Digestibility

The contents of dry matter (DM), crude protein (CP), ether extract (EE), neutral detergent fiber (NDF) and acid detergent fiber (ADF) in the experimental diet and feces samples were determined and the apparent nutrient digestibility was calculated using the method described in the previous study [17]. 

### 2.4. Blood Biochemical Indicators

The concentrations of serum glucose (GLU), triacylglycerols (TG), β-hydroxybutyric acid (BHBA), total cholesterol (TC), total protein (TP), and urea nitrogen (BUN) were analyzed by using an Automatic Biochemical Analyzer (SHIMADZU, Kyoto, Japan). Serum non-esterified fatty acid (NEFA) concentrations were determined by a colorimetry assay kit (Nanjing Jiangcheng, Jiangsu, China) referring to the manufacturer’s instructions.

### 2.5. Rumen Fermentation Parameters

The pH values of the rumen fluids were analyzed using a pH meter (INESA, Shanghai, China). Volatile fatty acids (VFA) in the rumen fluid were determined by gas chromatography (Agilent Technologies, Santa Clara, CA, USA) referring to Stewart and Duncan [18]. The ruminal microbial protein (MCP) concentration was determined by the method of Makkar et al. [19], and ammonia nitrogen concentrations were determined by alkaline sodium hypochlorite–phenol spectrophotometry [19].

### 2.6. Polymerase Chain Reaction Amplification and Sequencing

The total DNA of 36 ruminal fluid samples in experiment 2 were extracted using the TIANamp stool DNA kit (Tiangen Biotech., Beijing, China) according to the instructions that included a bead-beating step and subsequent DNA purification using a spin column. The quality and concentration of DNA were assessed using a NanoDrop Spectrophotometer (Thermo Scientific, Waltham, MA, USA). The V4 region of the bacterial 16 s rRNA gene was amplified using specific PCR primers (515F: 5’-GTGCCAGCMGCCGCGGTAA-3’ and 806R: 5’-GGACTACHVGGGTWTCTAAT-3’) contained barcode sequences. Three replicates of PCR reactions for each sample were combined. The quality of the PCR products was assessed by 2% agarose gel electrophoresis. An OMEGA Gel Extraction Kit (Omega Bio-Tek, Norcross, GA, USA) was used to extract and purify the DNA in the bright main strip between 200–400 bp. DNA was quantified using a Qubit@ 2.0 Fluorometer (Thermo Scientific, Waltham, MA, USA). The PCR products were pooled in equimolar amounts. The sequencing library was generated using a TruSeq DNA PCR-Free Sample Prep Kit following the manufacturer’s instructions. Then the samples were subjected to paired-end sequencing (2 × 250 bp) using an Illumina Hiseq apparatus at Rhonin Biosciences Co., Ltd.

### 2.7. Bioinformatics and Statistical Analysis

The paired-end raw reads were merged using FLASH [20] and assigned to each sample according to their unique barcodes. Data filtering was completed after removing low-quality bases by using Usearch, and high-quality target sequences were obtained for subsequent analysis. Sequences with an identity threshold ≥97% were clustered into the same Operational taxonomic units (OTUs) by using the UPARSE algorithm [21]. The OTUs with a read number >3 in at least one of the samples were used for the following analysis [22]. Representative sequences were picked and potential chimeras were removed using the Uchime algorithm [23]. Taxonomy was assigned by using the UCLUST classifier (identity threshold of 0.9) in the QIIME (version 1.8.0) and Silva 128 database [24]. The relative abundance of the bacterial taxon was normalized based on the OTU data. The alpha-diversity indexes, including Chao I, observed species, PD whole tree, and Shannon–Wiener, were calculated using Vegan R package. Principal component analysis (PCA) was used to reduce the dimensions of the rumen microflora data. The bacterial functional pathways were predicted using phylogenetic investigation of communities by reconstruction of unobserved states (PICRUSt) based on 16s DNA sequence data against the Greengenes reference taxonomy (release 13.5) and the Kyoto Encyclopedia of Genes and Genomes (KEGG) reference database [25].

All data were analyzed using SPSS 20.0 (SPSS Inc, Chicago, IL, USA). The statistical difference of normally distributed data, included growth performance, nutrients digestibility, blood biochemistry, and rumen fermentation, were analyzed by using independent sample T-test testing (differences between control and starvation group in experiment part 1) and One-way ANOVA followed Duncan’s posthoc testing (differences between time points in experiment 2). The 16 s rDNA sequencing data were non-normal and analyzed by using Kruskal–Wallis 1-way ANOVA following the posthoc all pairwise test. *p* values less than 0.05 were regarded as statistically significant. 

These sequence data were submitted to the NCBI database under accession number PRJNA550290.

## 3. Results

### 3.1. Experiment 1—Effects of Starvation and Refeeding on Yak Growth Performance

#### 3.1.1. Growth Performance and Nutrients Digestibility during Starvation and Refeeding Periods

**Figure 1:**
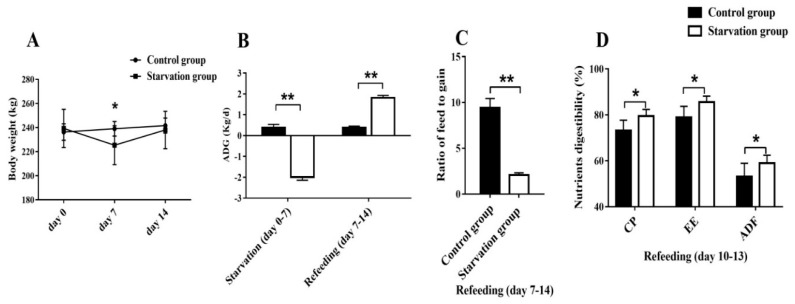
Effects of starvation and refeeding on the growth performance and nutrient digestibility of yaks. Comparisons of the body weight (**A**), ADG (average daily gain) (**B**), F/G (the ratio of feed to gain) (**C**) and nutrient digestibility (**D**) between the starvation and control groups. Values are means ± SD (*n* = 6). Differences were represented by * *p* < 0.05 and ** *p* < 0.01.

In experiment part 1, the 7-day starvation significantly reduced the body weight of yaks (*p* < 0.05) (Figure 1A). Refeeding after starvation significantly increased the ADG (*p* < 0.05), decreased the ratio of feed to gain (*p* < 0.05) and increased the digestibility of dietary CP, EE, and ADF (Only the significant data are shown here, *p* < 0.05) compared with the control group (Figure 1B,C).

#### 3.1.2. Blood Biochemistry during Starvation and Refeeding Periods

The results showed these biochemical blood indicators on day 0 had no significant difference between the starvation and control groups (*p* > 0.05). After the 7-day starvation, the serum concentrations of GLU, TG, and BUN in the starvation group were significantly lower than those in the control group (*p* < 0.05), whereas the serum concentrations of NEFA and BHBA in the starvation group were approximately 5.6-fold and 1.5-fold higher than those in the control group (*p* < 0.05). After the 7-day refeeding, these above-mentioned indicators recovered to similar levels to the control group (*p* > 0.05) (Table 2).

#### 3.1.3. Rumen Fermentation during Starvation and Refeeding Periods

**Figure 2:**
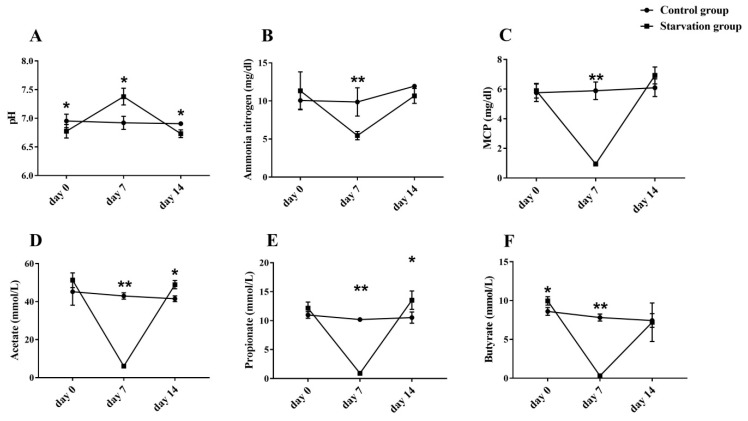
Effects of starvation and refeeding on the rumen fermentation parameters of yaks. (**A**) pH; (**B**) ammonia nitrogen (mg/dL); (**C**) MCP, microbial protein (mg/dL); (**D**) acetate (mmol/L); (**E**) propionate (mmol/L); (**F**) butyrate (mmol/L). Values are means ± SD (*n* = 6). Differences were represented by *p* < 0.05 and * *p* < 0.01.

The 7-day starvation significantly increased rumen pH and decreased concentrations of acetate, propionate, butyrate, MCP, and ammonia nitrogen of the starvation group (*p* < 0.05). After 7-day refeeding, the ruminal acetate and propionate concentrations of the starvation group were significantly higher than those of the control group (*p* < 0.05), meanwhile rumen MCP concentration of the starvation group trended higher than that of the control group (*p* = 0.054) (Figure 2).

### 3.2. Experiment 2—Effects of Starvation and Refeeding on Microbial Composition in the Rumen of Yaks

#### 3.2.1. Rumen Fermentation Alterations throughout the Normal Feeding, Starvation and Prolonged Refeeding Periods

**Figure 3:**
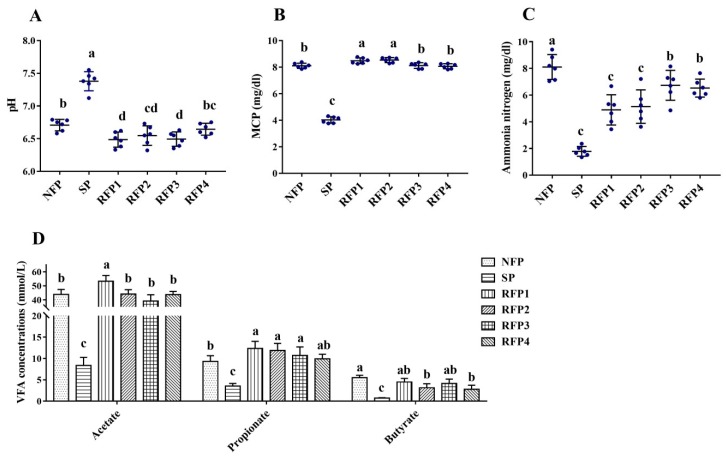
The fluctuations of microbial fermentation parameters, including pH (**A**), MCP (microbial protein) (**B**), ammonia nitrogen (**C**) and VFAs (volatile fatty acids) (**D**), of yaks through the normal feeding (NFP), starvation (SP) and prolonged refeeding (RFP1-4) periods. Values are means ± SD (*n* = 6). Different small letter superscripts represented significantly different (*p* < 0.05).

In experiment part 2, results also showed starvation significantly inhibited rumen microbial fermentation (*p* < 0.05). During the prolonged refeeding period, the rumen pH during RFP1 to RFP3 and the ammonia nitrogen concentration during the entire RFP (four weeks) were significantly lower than those of NFP, respectively (*p* > 0.05). Meanwhile, the acetate concentration of RFP1, the MCP concentrations of RFP1 and RFP2, and the propionate concentrations of RFP1 to RFP3 were significantly higher than those of NFP, respectively (*p* < 0.05) (Figure 3).

#### 3.2.2. Rumen Microflora Alterations throughout the Normal Feeding, Starvation and Prolonged Refeeding Periods

The PCA analysis showed that rumen fluid samples of SP had distinctly different microflora from the samples of NFP and RFP, and these differences gradually decreased with the prolonged refeeding time (Figure S1). After 7-day starvation, the α diversity indexes of rumen microflora, including the Chao I, Observed species and Shannon–Wiener, were significantly decreased (*p* < 0.05). During the refeeding period, the Chao 1, Observed species and Shannon–Wiener recovered gradually to similar levels of NFP in RFP3 (Table S1).

**Figure 4:**
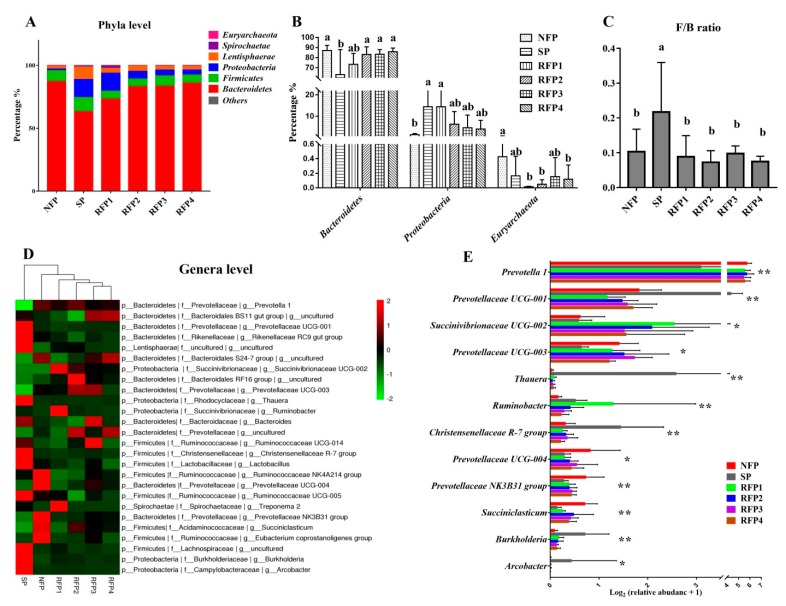
Starvation and prolonged refeeding remodel microflora in the rumen of yaks. (**A**) Rumen microbial community composition at phyla level. (**B**) The significantly changed bacteria at the phyla level. (**C**) The fluctuation of the F/B ratio (the ratio of Firmicutes to Bacteroidetes) through the experimental periods. (**D**) Rumen microbial community composition at genera level. (**E**) The significantly changed bacteria at the genera level. In the heatmap, the X-axis represents different groups, and the Y-axis represents different bacterial genus. The abundances of the bacterial genus are represented by the color intensity. Different small letter superscripts represent significantly different (*p* < 0.05). * represents *p* < 0.05, ** represents *p* < 0.01.

At the phyla level, the predominant bacterial populations (abundance >0.5% in at least one of the groups) through all periods are shown in Figure 4A, such as *Bacteroidetes* (63.24–87.00%), *Firmicutes* (5.88–11.23%), *Proteobacteria* (1.37–14.41%) and *Lentisphaerae* (2.00–9.83%). After 7-day starvation, the abundance of *Bacteroidetes* decreased significantly (*p* < 0.05), whereas the abundances of *Proteobacteria* and the ratio of *Firmicutes* to *Bacteroidetes* (F/B) increased significantly (*p* < 0.05). During the refeeding period, the abundance of *Bacteroidetes* in the RFP1 increased to a similar level to NFP, and the F/B in the RFP1 decreased to the same level as NFP (*p* > 0.05). The *Proteobacteria* population gradually reduced in the RFP2. Interestingly, the *Euryarchaeota* population trended toward reduction during the starvation period, and its abundance in the RPF1, RPF2 and RPF4 was continuously lower than that in the NFP (*p* < 0.05) (Figure 4B,C).

At the genus level, the predominant bacterial populations (abundance > 0.5% in at least one of the groups) are shown in Figure 4D, such as *Prevotella 1* (7.96–56.40%), *Prevotellaceae UCG 001* (1.31–24.33%), *Rikenellaceae RC9 gut group* (3.15–10.38%), *Succinivibrionaceae UCG 002* (0.53–8.40%) and *Prevotellaceae UCG 003* (0.57–2.43%). After 7-days starvation, the abundances of *Prevotella 1*, *Prevotellaceae UCG-003*, *Prevotellaceae UCG-004*, *Prevotellaceae NK3B31* group and *Succiniclasticum* significantly decreased (*p* < 0.05), whereas the abundances of *Prevotellaceae UCG-001*, *Thauera*, *Christensenellaceae R-7* group, *Burkholderia* and *Arcobacter* significantly increased (*p* < 0.05). During the refeeding period, the abundances of *Prevotella 1* and *Prevotellaceae UCG-003* in the RFP1 increased to a similar level as the NFP (*p* > 0.05). The abundances of *Prevotellaceae UCG-001*, *Thauera*, Christensenellaceae *R-7 group*, *Burkholderia*, and *Arcobacter* in the RFP1 decreased back to a similar level to NFP, respectively (*p* > 0.05). Interestingly, starvation had no significant effects on the *Succinivibrionaceae UCG-002* and *Ruminobacter* populations (*p* > 0.05), but they were both significantly increased in RFP1 (*p* < 0.05) and subsequently gradually decreased over RFP2 to RFP4 (Figure 4E and Table S2).

**Figure 5:**
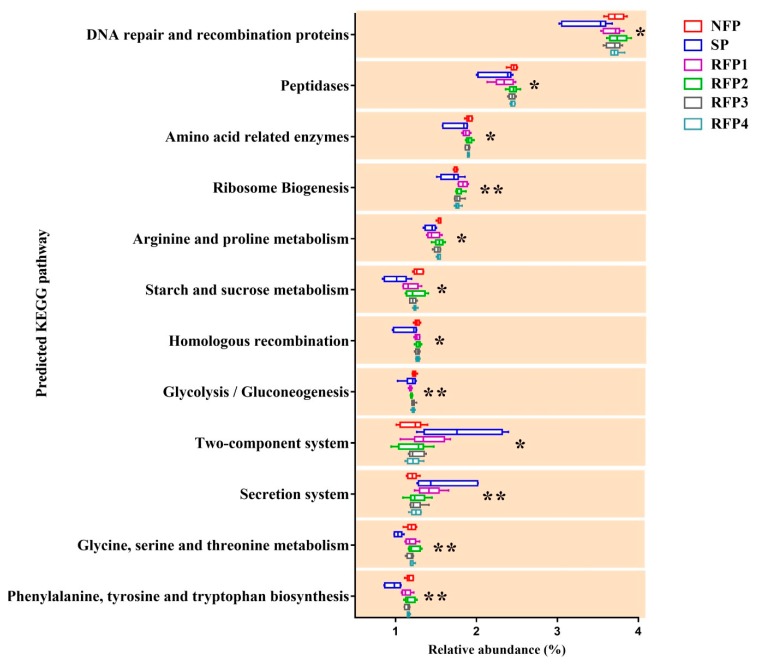
Comparisons of PICRUSt predicted functional pathways in the rumen microflora of yaks over the experimental periods (* *p* < 0.05, ** *p* < 0.01).

The PICRUSt predictive tool enriched 35 predominant pathways (abundance > 0.5% in at least one of the groups) at KEGG level 3 in the rumen microflora. Among them, 12 pathways were significantly affected through the starvation and prolonged refeeding periods (Figure 5, Table S3). Most of the functional pathways belonged to nutrients metabolism. These nutrient metabolic pathways, such as amino acid-related enzymes, arginine and proline metabolism, starch and sucrose metabolism, glycine, serine and threonine metabolism, and phenylalanine, tyrosine and tryptophan biosynthesis, significantly decreased in the starvation period and then increased to a peak in the RFP2 (*p* < 0.05). Additionally, the two-component system and secretion system pathways were both significantly highly represented in the starvation period (*p* < 0.05).

## 4. Discussion

Yaks unavoidably encounter severe starvation in the cold season and recover relatively rapid growth in the warm-season year after year, because of the harsh plateau environment. In winter, the most critical situation is heavy snow disaster, in which the land is covered by thick snow and yaks cannot access any forage for almost one week. Kirton et al. (1972) reported that beef cattle lost 10.2% of body weight after 4 days of pre-slaughter starvation [26]. Our previous studies found that with 3-year-old yak after 7 days starvation, the weight was significantly reduced by 10.06%, GLU reduced by 16.96%, NEFA increased by 21.70%, which shows that after 7 days starvation an energy negative balance of yak appears and reflects the typical seasonal food supply of yak [27]. Rumen microorganism ferments 70–80% of the feed intake and produces VFA to provide 60–75% of the required metabolic energy of the host [28]. Therefore, this study investigated the coping strategy of rumen microorganism of yaks for this natural challenge.

Weight loss is the most obvious and common response to starvation. In experiment part 1, the 7-day starvation dramatically reduced the body weight of yaks and decreased the serum glucose concentrations, the most crucial direct energy source of the animal, suggesting the body metabolism was in a negative energy balance. The significantly reduced serum TG level and increased NEFA level also proved that the fat tissue storage of yak was dramatically mobilized after the 7-day starvation [29]. Increasing lipolysis also produced BHBA, a typical energy source molecule for the animal during long-term starvation, which is consistent with our results. Additionally, the 7-day starvation significantly inhibited rumen microbial fermentation. The rumen acetate, propionate, butyrate, and MCP concentrations in the starvation group after starvation were only 14.1%, 8.8%, 3.8%, and 16.0% of those in the control group, respectively, suggesting that the rumen digesta was almost exhausted. Therefore, these results indicated that yaks were in the postabsorptive condition after 7-day starvation. 

Refeeding rapidly recovered the body weight of yaks with a significantly higher ADG. This may be due to the nutrient restriction during the starvation period, which resulted in compensating the growth of yaks during the refeeding period. Compensatory growth refers to the process of increasing the growth rate of animals after nutrient restriction [30]. Wilson and Osbourne (2010) report that the more severe the nutritional restriction, the faster the weight gain after supplementation [31]. Heifers whose feed intake was alternately 20% below or 25% resulted in improved efficiency of growth compared with controls (7.8% vs. 8.3%) [32]. After 7 days of fasting, yaks in the starvation group in this experiment lost 13.95 kg of weight, the daily weight gain during the refeeding period was 376.32% higher than the normal feeding period. The results of our other experiment found that with 3-year-old yaks after 7 days starvation, the weight is significantly reduced 19.5 kg, and the daily weight gain during the first week in the refeeding period increased by 361.54%, and then gradually decreased and did not differ significantly from the normal feeding period at the third week of the refeeding period [27]. Yambayamba et al. (1996) also found that cows can significantly improve feed efficiency and average daily weight gain after restricted feeding [33]. Our results also proved that yaks have higher digestibility of dietary CP, EE, and ADF, potentially contributing to the rapid ADG during the refeeding period. Fox et al. (1972) reported that the weight of castrated cattle increased from 260 kg to 350 kg at the initial stage of compensation, and the restricted group deposited more proteins than the control group [34]. This may be to make up for the loss of gastrointestinal tract, liver and other internal organs in the period of nutrient restriction, so as to increase protein deposition [35]. In addition, the increase of ADF digestibility in this study may be caused by changes in the rumen fermentation. The rumen is a major digestive organ for ruminants through microbial fermentation. Refeeding significantly increased the rumen acetate and propionate concentrations of yaks in the starvation group when compared to the control group. McCabe et al. (2015) reported that nutrient restriction and the return to ad libitum diet in beef cattle would change the rumen microbial composition [13]. Therefore, the changes in the concentration of volatile fatty acids in the rumen in this study may suggest the rumen microbial community compositions were potentially improved after starvation.

Therefore, experiment part 2 was conducted to deeply investigate the successive fluctuations of rumen microflora of yaks throughout the starvation and prolonged refeeding periods. Rumen pH is mainly affected by the production and absorption rate of VFA. The significantly increasing pH during the 7-day starvation suggested sharply attenuated microbial fermentation in the rumen. Then, the ruminal pH in the first 3 weeks of the refeeding period was continuously and significantly lower than that of NFP, even though the yaks were fed the same diet. This indicated the rumen microorganisms may produce more organic acids (VFAs) during the refeeding period. In particular, the results showed that rumen propionate concentration during the first 3 weeks of the refeeding period was continuously and significantly higher than that before starving. Rumen propionate fermentation has a higher dietary energy utilization efficiency than acetate and butyrate fermentation [13]. Thus, it indicated a significant improvement of microorganism harvesting energy from the diet during the refeeding period. Additionally, ruminal MCP can provide 40–80% of the protein requirements of the host [36]. In this study, rumen MCP concentration in the RFP1 and RFP2 was significantly higher than that in the NFP, indicating a higher efficiency of dietary nitrogen utilization by microorganisms in the first 2 weeks of the refeeding period. These fermentation parameters thus indicated that ruminal microorganisms were more efficient in fermenting dietary nutrition and provided more nutrients such as VFAs and MCP for the yaks during refeeding.

The 16s rDNA sequencing results showed that microbial community compositions in the rumen of yaks were significantly altered during starvation and prolonged refeeding stages. At the phyla level, the *Euryarchaeota* is mainly related to methane production in the rumen, and 2–12% of the total feed energy intake of ruminants is lost in the form of methane [37]. Previous studies reported that less grass available in the spring (April) and the relative abundance of *Methanobrevibacter* is higher in the rumen of yak, however in the summer (August) more grass is available, but the relative abundance of the *Methanobrevibacter* is lower in the rumen of yak on the Qinghai–Tibet Plateau [38]. Interestingly, our results showed that *Euryarchaeota* population significantly decreased at the beginning of refeeding compared with that before starvation, and the relative abundance *Methanobrevibacter* in Euryarchaeota significantly reduced (from 0.42 in NFP reduced to 0.01 in RFP1), indicating that refeeding after starvation might potentially reduce dietary energy waste in methane production in the rumen ecosystem. 

At the genera levels, three of the predominant microbes belonged to the *Prevotella* population. It has been previously reported that *Prevotella* is the most abundant genus in the rumen. Its primary function is to degrade protein and hemicellulose to produce acetate and propionate [39]. The primary function of *Succiniclasticum* in the rumen is to convert succinic acid into propionate to provide energy for the host [40]. Herein, we found that the abundances of *Prevotella 1*, *Prevotellaceae UCG-003*, *Prevotellaceae NK3B31* group and *Succiniclasticum* significantly reduced during starvation and recovered during refeeding, indicating starvation reduced the rumen microbial fermentation of predominant dietary nutrients. Additionally, the *Thauera*, *Christensenellaceae R-7*group, *Burkholderia* and *Arcobacter* populations dramatically increased during starvation, of which the *Thauera* population increased about 180-fold compared to before starvation and after refeeding. Most strains of the *Thauera* genus belonged to the *Betaproteobacteria* phylum and it was the typical denitrifying bacteria, which fermented nitrate and other nitrogen oxides to ammonium ion or nitrogen [41]. Our results suggested that the nitrogen metabolism potentially changed in the rumen ecosystem to adapt to starvation. Interestingly, the *Succinivibrioanceae UCG-001* and *Ruminobacter* populations had no significant changes during starvation, but they dramatically increased at the beginning of the refeeding period. McCabe et al. (2015) reported that after 125 days of nutritional restriction (0.6 kg/d per head), the abundance of Succinivibrionaceae in the rumen of beef cattle decreased significantly, and its abundance of 55-day-re-feeding (ad libitum) increased, and it was not significantly different from the control group [13]. The *Succinivibrioanceae* compete with methanogens for hydrogen as a substrate to production succinate and propionate rather than methane and thus improve the energy utilization efficiency [42]. The *Ruminobacter amylophilus* is the major *Ruminobacter* in the rumen, which is mainly responsible for digesting starch and producing acetate and propionate [43]. These bacterial population changes were in accordance with the results that rumen acetate and propionate concentrations during refeeding were higher than those before starvation, suggesting higher efficiencies of nutrient harvesting in the rumen ecosystem at the beginning of the refeeding period.

We analyzed the potential function pathways in the rumen microflora of yaks by using PICRUSt. Most of the microbial nutrient metabolic pathways were inhibited during the starvation periods, because the fermentation substrates reduced in the rumen. Interestingly, the results showed that most of the nutrient metabolic pathways, mainly related to amino acid, starch, and sucrose metabolism, were increased back to a peak in the second week of the refeeding period, suggesting the rumen microflora may obtain an efficient nutrient metabolism in the second week of refeeding. 

## 5. Conclusions

Overall, the present data showed that starvation and re-alimentation altered the metabolism and growth rate of yaks. These changes were closely related to the adaptability of yak rumen microorganisms. Re-alimentation after nutritional deprivation improved the feed efficiency, rumen microbial fermentation, and microbial community compositions of yak, that led the yak to have higher energy and protein utilization efficiency during the refeeding period after starvation. These alterations, in part, contribute to yak adaption to starvation and re-alimentation. Therefore, nutritional regulation may be used to promote the body weight of starved yak to recover quickly in future yak management in the cold season of the Qinghai–Tibetan Plateau, such as timely provision of energy and protein-rich diets after starvation stress.

## Figures and Tables

**Table 1:** Composition and nutrient levels of the experimental diet (air-dry basis, %).

Items		Content
Ingredients	Corn	20.30
	Wheat bran	6.30
	Soybean meal	2.80
	Rapeseed meal	3.50
	Distilled grains	35.00
	Rice straw	30.00
	CaHPO_4_	0.77
	CaCO_3_	0.04
	NaHCO_3_	0.42
	NaCl	0.52
	Premix ^1^	0.35
	Total	100.00
Nutrient levels	Dry Matter (DM)	90.73
	NEmf (MJ/kg) ^2^	5.10
	Crude Protein (CP)	12.56
	Neutral Detergent Fiber (NDF)	54.14
	Acid Detergent Fiber (ADF)	41.05
	Ca	0.73
	Total phosphorus (TP)	0.40

^1^ The premix provided the following per kg of the diet: Co 12 mg, Cu 11.67 mg, I 0.58 mg, Fe 58.33 mg, Mn 23.33 mg, Se 0.23 mg, Zn 35 mg, Vitamin A 3.00 × 104 IU, Vitamin D 1.20 × 104 IU, Vitamin E 90.00 IU. ^2^ NEmf was the net energy for maintain and fattening, which was calculated reference to the equations on the Chinese Feeding standard of beef cattle (NY/T 815-2004).

**Table 2:** Effects of starvation and refeeding on the blood biochemical indices of yak.

Items	Periods	Groups		SEM	*p*-Value
		Control Group	Starvation Group		
GLU (mmol/L)	Day 0	3.89	3.94	0.48	0.779
	Day 7	3.84 ^a^	3.19 ^b^	0.53	0.029
	Day 14	3.63	3.46	0.47	0.409
BHBA (mmol/L)	Day 0	0.28	0.28	0.06	0.719
	Day 7	0.26 ^b^	0.39 ^a^	0.08	0.01
	Day 14	0.25	0.24	0.05	0.855
TG (mmol/L)	Day 0	0.28	0.26	0.06	0.506
	Day 7	0.28 ^a^	0.17 ^b^	0.04	0.001
	Day 14	0.25	0.19	0.06	0.056
TC (mmol/L)	Day 0	1.18	1.15	0.41	0.865
	Day 7	1.26	1.46	0.44	0.319
	Day 14	1.11	1.25	0.38	0.425
NEFA (mmol/L)	Day 0	0.23	0.23	0.10	0.979
	Day 7	0.18 ^b^	1.01 ^a^	0.31	0.001
	Day 14	0.26	0.22	0.10	0.675
BUN (mmol/L)	Day 0	4.27	4.01	0.28	0.072
	Day 7	4.44 ^a^	3.63 ^b^	0.27	0.001
	Day 14	4.49 ^a^	2.67 ^b^	0.46	0.000
TP (g/L)	Day 0	64.18	65.55	7.96	0.692
	Day 7	63.53	61.20	8.42	0.527
	Day 14	64.10	66.25	11.05	<0.000

SEM: standard error of the mean. Abbreviations: GLU, Glucose; BHBA, β-hydroxybutyric acid; TG, triglyceride; TC, total cholesterol; NEFA, non-esterified fatty acid; BUN, blood urea nitrogen; TP, total protein. ^a,b^ Different small letter superscripts represent significant difference (*p* < 0.05).

## Supplementary Materials

The following are available online at https://www.mdpi.com/2076-2615/9/10/807/s1, Figure S1: The PCA scores plot of rumen microflora at different periods, Table S1: Alpha diversity of rumen microflora of yaks through the experimental periods, Table S2: Effects of starvation and prolonged refeeding on the relative abundances of ruminal bacteria (%) at the genus level in yaks, Table S3: Effects of starvation and prolonged refeeding on the relative abundances of predominant microbial metabolic pathways (%).
