# Supplementary material for: Effects of Nutritional Deprivation and Re-Alimentation on the Feed Efficiency, Blood Biochemistry, and Rumen Microflora in Yaks (Bos grunniens)
Source: Animals (Basel). 2019 Oct 15;9(10):807. doi: 10.3390/ani9100807 (PMC6826638; doi:10.3390/ani9100807)
Supplement: Supplementary file 1 [file animals-09-00807-s001.pdf]

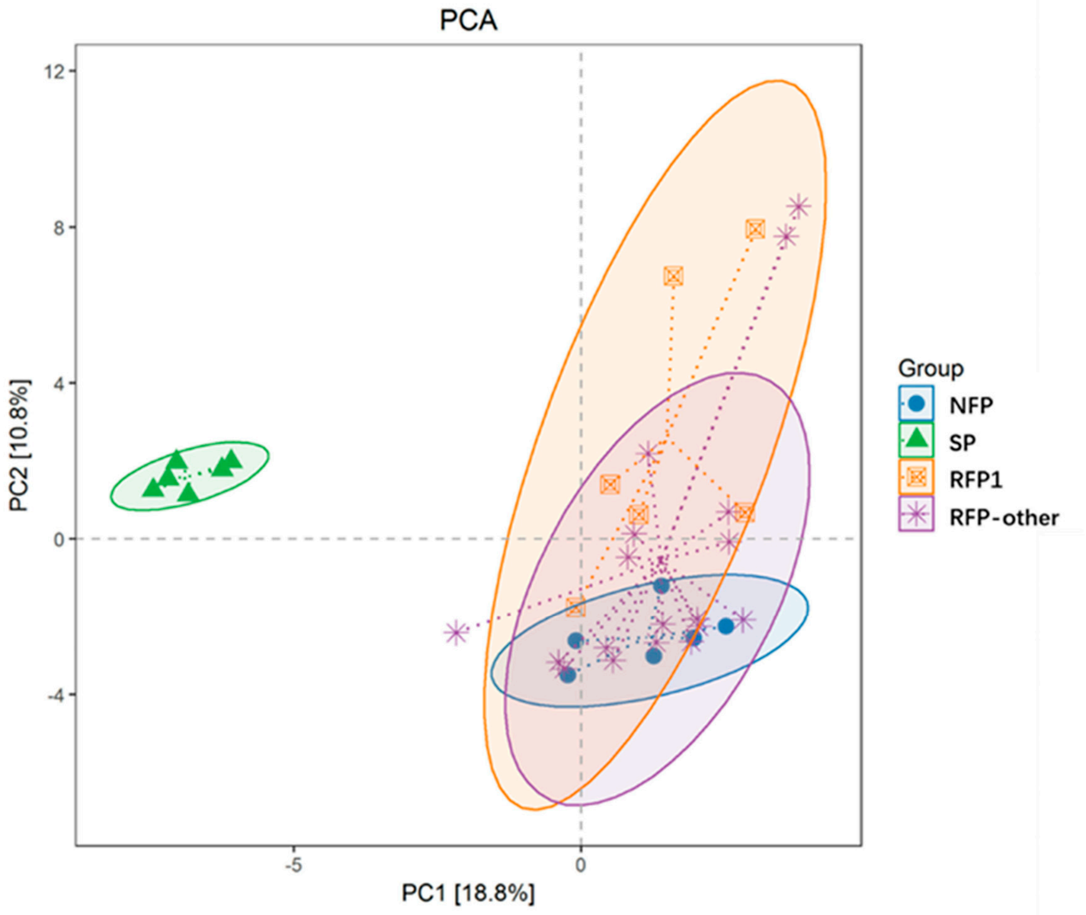


**Figure S1.** The PCA scores plot of rumen microflora at different periods.

**Table S1.** Alpha diversity of rumen microflora of yaks through the experimental periods.

| Items | Groups | | | | | | SEM | *p* |
|---|---|---|---|---|---|---|---|---|
| | NFP | SP | RFP1 | RFP2 | RFP3 | RFP4 | | |
| Chao Ⅰ | 2072.77 [b] | 1956.52 [c] | 1991.61 [c] | 1831.00 [d] | 2199.50 [a] | 2192.12 [a] | 23.79 | 0.000 |
| Observed species | 1449.5 [a] | 1240.33 [b] | 1244.33 [b] | 1263.00 [b] | 1456.67 [a] | 1407.00 [a] | 21.40 | 0.000 |
| PD whole tree | 95.63 [a] | 96.61 [a] | 88.93 [b] | 88.68 [b] | 98.75 [a] | 96.24 [a] | 0.87 | 0.000 |
| Shannon-wiener | 5.36 [a] | 4.37 [b] | 4.61 [b] | 4.63 [b] | 5.18 [a] | 5.08 [a] | 0.07 | 0.000 |

Data with different small letter superscripts within the same row were significantly different ($p < 0.05$). NFP, normal feeding period; SP, starvation period; RFP1, 1st week of refeeding period; RFP2, 2nd week of refeeding period; RFP3, 3rd week of refeeding period; RFP4, 4th week of refeeding period. SEM: standard error of the mean.

**Table S2.** Effects of starvation and prolonged refeeding on the relative abundances of ruminal bacteria (%) at the genus level in yaks.

| Items | Groups | | | | | | SEM | $p$ |
|---|---|---|---|---|---|---|---|---|
| | NFP | SP | RFP1 | RFP2 | RFP3 | RFP4 | | |
| *Prevotella 1* | 55.03 $^{a}$ | 7.96 $^{b}$ | 48.58 $^{a}$ | 56.40 $^{a}$ | 46.49 $^{a}$ | 48.97 $^{a}$ | 3.607 | 0.009 |
| *Prevotellaceae UCG-001* | 2.72 $^{b}$ | 24.33 $^{a}$ | 1.31 $^{b}$ | 1.85 $^{b}$ | 2.27 $^{b}$ | 2.35 $^{b}$ | 1.848 | 0.001 |
| *Succinivibrionaceae UCG-002* | 0.63 $^{b}$ | 0.53 $^{b}$ | 8.40 $^{a}$ | 4.83 $^{ab}$ | 3.56 $^{ab}$ | 3.10 $^{ab}$ | 0.913 | 0.030 |
| *Prevotellaceae UCG-003* | 1.77 $^{ab}$ | 0.57 $^{b}$ | 1.57 $^{ab}$ | 2.43 $^{a}$ | 2.41 $^{a}$ | 1.33 $^{ab}$ | 0.213 | 0.016 |
| *Thauera* | 0.03 $^{b}$ | 9.13 $^{a}$ | 0.06 $^{b}$ | 0.04 $^{b}$ | 0.04 $^{b}$ | 0.05 $^{b}$ | 0.892 | 0.003 |
| *Ruminobacter* | 0.12 $^{b}$ | 0.45 $^{b}$ | 4.62 $^{a}$ | 0.35 $^{b}$ | 0.23 $^{b}$ | 0.18 $^{b}$ | 0.686 | 0.010 |
| *Christensenellaceae R-7 group* | 0.25 $^{b}$ | 2.28 $^{a}$ | 0.20 $^{b}$ | 0.26 $^{b}$ | 0.29 $^{b}$ | 0.17 $^{b}$ | 0.109 | 0.004 |
| *Prevotellaceae UCG-004* | 0.94 $^{a}$ | 0.23 $^{b}$ | 0.23 $^{b}$ | 0.33 $^{ab}$ | 0.52 $^{ab}$ | 0.37 $^{ab}$ | 0.016 | 0.046 |
| *Prevotellaceae NK3B31 group* | 0.72 $^{a}$ | 0.22 $^{b}$ | 0.31 $^{b}$ | 0.32 $^{b}$ | 0.37 $^{b}$ | 0.36 $^{b}$ | 0.042 | 0.005 |
| *Succiniclasticum* | 0.66 $^{a}$ | 0.11 $^{c}$ | 0.19 $^{bc}$ | 0.45 $^{ab}$ | 0.35 $^{bc}$ | 0.31 $^{bc}$ | 0.046 | 0.004 |
| *Burkholderia* | 0.07 $^{b}$ | 0.73 $^{a}$ | 0.13 $^{b}$ | 0.12 $^{b}$ | 0.10 $^{b}$ | 0.11 $^{b}$ | 0.056 | 0.007 |
| *Arcobacter* | 0.00 $^{b}$ | 0.70 $^{a}$ | 0.01 $^{b}$ | 0.01 $^{b}$ | 0.00 $^{b}$ | 0.00 $^{b}$ | 0.109 | 0.026 |

The significantly changed predominant genera populations (abundance > 0.5% in at least one of the groups) were shown. Data with different small letter superscripts within the same row were significantly different ($p < 0.05$).NFP, normal feeding period; SP, starvation period; RFP1, 1st week of refeeding period; RFP2, 2nd week of refeeding period; RFP3, 3rd week of refeeding period; RFP4, 4th week of refeeding period. SEM: standard error of the mean.

**Table S3.** Effects of starvation and prolonged refeeding on the relative abundances of predominant microbial metabolic pathways (%).

| Items | Groups | | | | | | SEM | $p$ |
|---|---|---|---|---|---|---|---|---|
| | NFP | SP | RFP1 | RFP2 | RFP3 | RFP4 | | |
| DNA repair and recombination proteins | 3.72 $^{a}$ | 3.40 $^{b}$ | 3.69 $^{a}$ | 3.75 $^{a}$ | 3.70 $^{a}$ | 3.71 $^{a}$ | 0.030 | 0.046 |
| Peptidases | 2.46 $^{a}$ | 2.28 $^{b}$ | 2.34 $^{ab}$ | 2.45 $^{a}$ | 2.44 $^{a}$ | 2.45 $^{a}$ | 0.020 | 0.049 |
| Amino acid related enzymes | 1.91 $^{a}$ | 1.77 $^{b}$ | 1.87 $^{ab}$ | 1.91 $^{a}$ | 1.89 $^{ab}$ | 1.90 $^{ab}$ | 0.013 | 0.032 |
| Ribosome Biogenesis | 1.75 $^{b}$ | 1.69 $^{b}$ | 1.83 $^{a}$ | 1.78 $^{ab}$ | 1.77 $^{ab}$ | 1.77 $^{ab}$ | 0.012 | 0.008 |
| Arginine and proline metabolism | 1.55 $^{a}$ | 1.44 $^{b}$ | 1.46 $^{ab}$ | 1.54 $^{ab}$ | 1.52 $^{ab}$ | 1.53 $^{ab}$ | 0.010 | 0.015 |
| Starch and sucrose metabolism | 1.28 $^{a}$ | 1.01 $^{b}$ | 1.18 $^{a}$ | 1.24 $^{a}$ | 1.22 $^{a}$ | 1.24 $^{a}$ | 0.020 | 0.012 |
| Homologous recombination | 1.27 $^{a}$ | 1.15 $^{b}$ | 1.27 $^{a}$ | 1.28 $^{a}$ | 1.27 $^{a}$ | 1.27 $^{a}$ | 0.012 | 0.050 |
| Glycolysis / Gluconeogenesis | 1.23 $^{a}$ | 1.19 $^{b}$ | 1.18 $^{b}$ | 1.20 $^{ab}$ | 1.22 $^{a}$ | 1.22 $^{a}$ | 0.006 | 0.004 |
| Two-component system | 1.21 $^{b}$ | 1.81 $^{a}$ | 1.38 $^{ab}$ | 1.23 $^{b}$ | 1.25 $^{b}$ | 1.22 $^{b}$ | 0.053 | 0.049 |
| Secretion system | 1.21 $^{b}$ | 1.58 $^{a}$ | 1.42 $^{ab}$ | 1.26 $^{ab}$ | 1.25 $^{ab}$ | 1.25 $^{ab}$ | 0.034 | 0.008 |
| Glycine, serine and threonine metabolism | 1.19 $^{a}$ | 1.03 $^{b}$ | 1.19 $^{ab}$ | 1.22 $^{a}$ | 1.18 $^{ab}$ | 1.20 $^{a}$ | 0.013 | 0.010 |
| Phenylalanine tyrosine and tryptophan biosynthesis | 1.17 $^{a}$ | 0.97 $^{b}$ | 1.13 $^{ab}$ | 1.17 $^{a}$ | 1.15 $^{ab}$ | 1.16 $^{a}$ | 0.015 | 0.006 |

The significantly changed predominant functional pathways (abundance > 0.5% in at least one of the groups) were shown. Data with different small letter superscripts within the same row were significantly different ($p < 0.05$). NFP, normal feeding period; SP, starvation period; RFP1, 1st week of refeeding period; RFP2, 2nd week of refeeding period; RFP3, 3rd week of refeeding period; RFP4, 4th week of refeeding period. SEM: standard error of the mean.
